# Tissue-Specific Expression of the Low-Affinity IgG Receptor, FcγRIIb, on Human Mast Cells

**DOI:** 10.3389/fimmu.2018.01244

**Published:** 2018-06-06

**Authors:** Oliver T. Burton, Alexandra Epp, Manoussa E. Fanny, Samuel J. Miller, Amanda J. Stranks, Jessica E. Teague, Rachael A. Clark, Matt van de Rijn, Hans C. Oettgen

**Affiliations:** ^1^Division of Immunology, Boston Children’s Hospital, Harvard Medical School, Boston, MA, United States; ^2^Department of Dermatology, Brigham and Women’s Hospital, Harvard Medical School, Boston, MA, United States; ^3^Department of Pathology, Stanford University Medical Center, Palo Alto, CA, United States

**Keywords:** IgE, IgG, FcγRIIb, Fc receptors, mast cells, anaphylaxis, immediate hypersensitivity, food allergy

## Abstract

Immediate hypersensitivity reactions are induced by the interaction of allergens with specific IgE antibodies bound *via* FcεRI to mast cells and basophils. While these specific IgE antibodies are needed to trigger such reactions, not all individuals harboring IgE exhibit symptoms of allergy. The lack of responsiveness seen in some subjects correlates with the presence of IgG antibodies of the same specificity. In cell culture studies and *in vivo* animal models of food allergy and anaphylaxis such IgG antibodies have been shown to exert suppression *via* FcγRIIb. However, the reported absence of this inhibitory receptor on primary mast cells derived from human skin has raised questions about the role of IgG-mediated inhibition of immediate hypersensitivity in human subjects. Here, we tested the hypothesis that mast cell FcγRIIb expression might be tissue specific. Utilizing a combination of flow cytometry, quantitative PCR, and immunofluorescence staining of mast cells derived from the tissues of humanized mice, human skin, or in fixed paraffin-embedded sections of human tissues, we confirm that FcγRIIb is absent from dermal mast cells but is expressed by mast cells throughout the gastrointestinal tract. IgE-induced systemic anaphylaxis in humanized mice is strongly inhibited by antigen-specific IgG. These findings support the concept that IgG, signaling *via* FcγRIIb, plays a physiological role in suppressing hypersensitivity reactions.

## Introduction

Clinical evaluation for allergy currently centers on detection of allergen-specific IgE antibodies using both skin testing and serum IgE measurements. The presence of specific IgE in patients with histories of allergen-induced reactions is considered confirmatory of allergy. However, while specific IgE is necessary for immediate hypersensitivity reactions, it is not always sufficient. This is particularly true in the setting of food allergy where a significant fraction of patients harboring allergen-specific IgE antibodies to foods can actually ingest those foods with no reaction (1–3). As a result, accurate diagnosis of food allergy can in many cases only be established by ingestion challenge. This poor predictive value of IgE testing offers an important clue regarding the regulation of IgE-triggered mast cell responses to allergens, suggesting the presence of counteracting mechanisms or inhibitory pathways in individuals producing significant amounts of allergen-specific IgE who are not clinically allergic.

Allergen-specific IgG antibodies can exert such a dampening effect on IgE-mediated responses. Epidemiological studies of cohorts of children have revealed that the prevalence of allergen-specific IgE responses to aeroallergens significantly exceeds that of symptomatic respiratory allergy and that the levels of allergen-specific IgG antibodies correlate with protection from symptoms (4). There is growing evidence for a similar benefit of IgG antibodies in food allergy. Specific IgG levels are inversely correlated with reaction severity in food allergic subjects and increase in parallel with the natural resolution of milk and egg allergy (5–7). Oral immunotherapy (OIT) and early food introduction strategies both elicit food-specific IgG responses (8–14). IgG has been shown to reduce IgE-mediated mast cell activation *via* two distinct mechanisms, (1) antigen interception and steric blockade, blocking binding to IgE or (2) *via* Fc-mediated interactions with the inhibitory receptor FcγRIIb (15). The importance of these IgG pathways in exerting suppression of hypersensitivity *in vivo* has been explored in murine studies in which it has been clearly demonstrated that both are at work but that FcγRIIb ligation is about an order of magnitude more potent in mediating IgE responses than is steric blockade (16–20).

Fcγ receptors (FcγRs) can be classified into activating and inhibitory FcγRs. Mouse mast cells express the activating receptor FcγRIII, while human mast cells express FcγRI and FcγRIIa, but not the low-affinity receptor, FcγRIII. The activating FcγRs, like the high-affinity IgE-receptor FcεRI, signal *via* a cytosolic immunoreceptor tyrosine-based activation motif (ITAM). Upon activation, the ITAMs are transphosphorylated, and a signaling cascade is initiated by the SH2-containing Syk tyrosine kinase. The receptor FcγRIIb is unique as it is the only inhibitory FcγR. It contains an immunoreceptor tyrosine-based inhibitory motif that recruits phosphatases for inhibitory and immunomodulatory downstream signaling. Thus, FcγRIIb is able to attenuate signaling induced by activating FcγRs (21–23). Murine mast cells express FcγRIIb, and genetic models have established that IgG-mediated suppression of IgE-induced anaphylaxis is dependent on its presence (16–19, 24).

The role of FcγRIIb in the suppression of human mast cell activation by IgE *in vivo* has been less clear. Like murine mast cells, human mast cells cultured from hematopoietic progenitors express functional FcγRIIb (25). In contrast, when isolated from the skin, the most accessible tissue from which to obtain them, primary human mast cells lack the receptor (26). This finding along with the observation that subjects who successfully complete food OIT do not exhibit anaphylaxis upon ingestion challenge despite having quite elevated IgE levels but still exhibit positive skin test responses to the same food (27–30) led us to hypothesize that IgG antibodies formed in the course of OIT might suppress the IgE-induced activation of intestinal mast cells (and hence food anaphylaxis) while leaving IgE-induced skin responses unchecked. A corollary of this hypothesis would be that intestinal but not cutaneous mast cells express FcγRIIb. Notably, allergen-specific IgG levels increase by orders of magnitude during OIT (27, 30, 31), and this IgG suppresses basophil degranulation in an FcγRII-dependent manner (18).

In order to test our hypothesis, we used an array of approaches to evaluate the expression of the low-affinity inhibitory Fc receptor, FcγRIIb, in human IgE receptor-bearing cells. We analyzed live cells isolated from human skin and various tissues of humanized mice as well as arrays of fixed tissues from a number of human organs. Our analyses confirm the previously reported absence of FcγRIIb in human skin mast cells but demonstrate its presence in mast cells of the gastrointestinal tract. Using the humanized mouse model, we demonstrate that IgG antibodies suppress IgE-triggered human mast cell-mediated anaphylaxis in an FcγRII-dependent manner.

## Materials and Methods

### Humanized Mice

Humanized mice with robust reconstitution both of human T and B cell adaptive immune compartments and human mast cells were produced as previously described (32, 33). Briefly, non-obese diabetic (NOD).SCIDγc^−/−^ mice transgenic for membrane-bound human stem cell factor (SCF) [NOD.Cg-*Prkdc^scid^Il2rg^tm1Wjl^* Tg(PGK1-KITLG*220)441Daw/SzJ] were engrafted with 5 × 10^4^ CD34^+^ hematopoietic stem cells (HSC) from cord blood (AllCells) for 16–24 weeks. Wild-type BALB/c, C57BL/6J, and FcγRIIb^−/−^ (B6) mice were bred at Boston Children’s Hospital. All animal work was conducted under protocols approved by the Institutional Animal Care and Use Committee at Boston Children’s Hospital.

### Cell Isolation

Peripheral blood was collected from healthy adult volunteers by venipuncture. Neonatal foreskins were obtained through the Human Skin Disease Resource Center. Cells were dispersed from the spleen and bone marrow of (humanized) mice by mechanical disruption. Leukocyte suspensions were prepared from skin and intestine according to established procedures using collagenase digestion (34). Human mast cells were isolated by immunomagnetic selection using CD117 microbeads (Miltenyi Biotec). Human mast cell progenitors were similarly isolated from humanized mouse bone marrow. Human basophils were isolated from Ficoll-separated human peripheral blood mononuclear cells using a Basophil Diamond Isolation Kit (Miltenyi Biotec).

### Cell Culture

Cells were cultured in RPMI-1640 medium supplemented with 10% heat-inactivated fetal calf serum, 1 mM sodium pyruvate, 1% MEM non-essential amino acids, 2 mM l-glutamine, 100 µg/ml streptomycin, 100 U/ml penicillin, 10 µg/ml gentamicin, 55 µM 2-mercaptoethanol, and 10 mM HEPES buffer (complete RPMI). Isolated human mast cells were cultured in the presence of human SCF (20 ng/ml, Shenandoah Biotechnology). Mast cell phenotypes were assessed by flow cytometry (c-Kit^+^FcεRIα^+^) and chloroacetate esterase staining.

### RNA Analysis

RNA was extracted using an RNeasy Micro Kit from Qiagen, reverse transcribed using a BioRad iScript cDNA synthesis kit, and analyzed by qPCR using TaqMan probes. GAPDH was used for normalization, and data are expressed as transcripts per 1,000 GAPDH transcripts.

### Immunofluorescence on Formalin-Fixed Paraffin-Embedded (FFPE) Tissues

Human tissue arrays were produced as previously described (35, 36). Paraffin-embedded tissue sections were deparaffinized according to standard procedures, and epitopes retrieved by heating for 20 min in 10 mM sodium citrate pH 6 with 0.05% Tween-20 in a vegetable steamer. Sections were blocked and permeabilized using 0.3% Triton X-100 with 2% BSA and 5% goat serum (all Sigma-Aldrich). The following antibodies were used: anti-human tryptase (clone AA1, mouse IgG1, Santa Cruz Biotechnology), anti-CD32B (rabbit polyclonal ab151497, Abcam), goat anti-mouse IgG1 AlexaFluor488 (Invitrogen), goat anti-mouse IgG2b AlexaFluor568 (Invitrogen), and goat anti-rabbit AlexaFluor568 (Invitrogen). Sections were mounted in Prolong Gold Antifade Reagent with DAPI (ThermoFisher) and imaged on a Nikon E800 microscope.

### Flow Cytometry

Cells were stained for surface markers in FACS buffer (PBS, 0.5% BSA, 0.01% sodium azide) at 4°C for 30 min. Peripheral blood was subjected to fixation and erythrocyte lysis using BD Phosflow Lyse/Fix reagent for 10 min. All other cells were fixed using BD Cytofix/Cytoperm reagent. After fixation, cells were washed and stained in BD permeabilization buffer.

Non-specific binding was blocked using FcX TruStain (Biolegend) and 10% rabbit serum (Sigma Aldrich). Cells were stained to detect CD45 (clone HI30, Biolegend), CCR3 (clone 5E8, Biolegend), c-Kit (clone 104D2, Biolegend), FcεRIα (clone CRA-1, Biolegend), CD64 (FcγRI) (clone 10.1, Biolegend), CD32A (FcγRIIa) (clone IV.3, Stem Cell Technologies), CD16 (FcγRIII) (clone 3G8, Biolegend), and viability (eBioscience Fixable Viability dyes). CD32B (FcγRIIb) was detected by a rabbit polyclonal antibody directed against the c-terminal (intracellular) portion of CD32B (Abcam, ab151497). This antibody was purified and directly conjugated to PE-Cy7 using a Lightning Link kit (Innova Biosciences). Non-specific rabbit IgG was treated identically. Specific staining for CD32B was accomplished using extensive blocking with unlabeled rabbit IgG (100 µg/ml, 15 min) and validated by comparing the signal obtained on B cells (CD32B^+^) versus T cells (CD32B^−^) (Figure S1 in Supplementary Material).

### IgG Preparation

IgG was prepared from pooled sera from de-identified peanut-allergic patients that had undergone OIT. IgG was purified over Nab protein G spin columns (Thermo Scientific), concentrated, and dialyzed with Macrosep Advance Centrifugal Devices carrying a 50 kDa cutoff (Pall Corporation) and filter-sterilized with 0.2 µM syringe filters (Millex). Allergen-specific IgG concentrations were determined by Phadia ImmunoCAP (ThermoFisher).

### Passive Anaphylaxis

Humanized mice were passively sensitized to peanut by intravenous injection of IgG-depleted serum from highly peanut allergic human donors, containing 70 ng αPN IgE as determined by ImmunoCAP. 24 h prior to allergen challenge, human IgG against PN was injected i.p. Anaphylaxis was evoked by i.p. injection of 1 mg complete peanut extract, which was made as previously described (18). Anaphylactic severity was determined by monitoring the loss of core body temperature using subdermally implanted microchip transponders (Bio Medic Data Systems).

### Statistical Analysis

Data were plotted and analyzed in Prism 5.0f (GraphPad Software, Inc.). Anaphylaxis data were analyzed using repeated measures two-way ANOVA; all other data were analyzed using standard ANOVA with Bonferroni post-tests between groups. ELISA values for IgE varied across multiple orders of magnitude and thus were subjected to log transformation prior to statistical analysis; for this purpose, null values were assigned a nominal value corresponding to the limit of detection in the assay. Two-tailed *P* values are summarized as follows: *P < 0.05, **P < 0.01, ***P < 0.001, and ****P < 0.0001. Data are represented as mean ± SEM for anaphylaxis curves, and with points showing individual mice overlaid with mean ± SEM elsewhere.

## Results

### Human Mast Cells Express Both Inhibitory and Activating FcγRII

Murine models have revealed the impact of the inhibitory Fc receptor FcγRIIb on pathogenesis of both allergic and autoimmune diseases (16–18). IgG signaling *via* FcγRIIb potently inhibits IgE-induced anaphylactic shock (16, 19, 24). Since mast cells are thought to be the main drivers of this IgE-mediated hypersensitivity reaction, the mechanisms by which their activation can be modulated by IgG are likely relevant for allergic disease. While it is established that murine mast cells, murine basophils, and human basophils all express high levels of inhibitory FcγRIIb (37, 38), human tissue mast cells are harder to obtain and are less well characterized in this regard. Previous observations that mast cells isolated from human skin express only the activating FcγR, FcγRIIa, whereas mast cells cultured from cord blood have only inhibitory FcγRIIb have suggested potential heterogeneity among human mast cells with respect to the expression of these receptors (25, 26).

**Figure 1:**
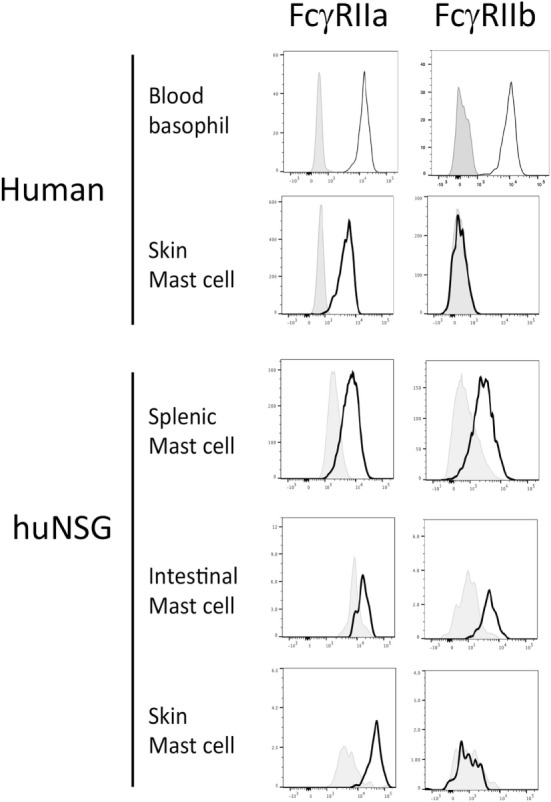
Fcγ receptor (FcγR)IIb expression by mast cells and basophils from humanized mice, human blood and human skin. Cells from human samples or humanized mouse organs were assessed for *FcgRIIa* and *FcgRIIb* expression by flow cytometry. Each experiment was performed at least three times and a representative histogram is presented.

Several humanized mouse models have been shown to foster human mast cell development and we reasoned that examination of primary cells from such mice might provide insight into the tissue-specific expression of Fc receptors (32, 33, 39, 40). For this analysis, we used non-obese diabetic (NOD) severe combined immunodeficient (SCID) mice lacking the cytokine receptor common gamma chain (γc^−/−^) and carrying a human SCF transgene that were engrafted with human HSC. We have previously described the engraftment of both a functional adaptive (T and B cell) human immune response as well as abundant human mast cells capable of mediating allergen-specific immediate hypersensitivity responses (33). Leukocytes were isolated from the tissues of such mice and flow cytometric analysis performed to measure FcγR expression on cells positive for c-Kit and human FcεRIα using both an antibody directed at an FcγRIIa (CD32A)-specific surface epitope as well as a peptide-specific polyclonal antibody targeting a sequence unique to the intracellular portion of FcγRIIb (CD32B) (Figure S1 in Supplementary Material). Consistent with our hypothesis and some prior observations, inhibitory FcγRIIb was expressed by mast cells from the intestine and spleen, but not from the skin of humanized mice (Figure 1). FcγRIIa was expressed by all mast cells.

Analysis of primary cells prepared from human skin samples confirmed that skin mast cells express FcγRIIa but not FcγRIIb (Figure 1). In contrast, human peripheral blood basophils expressed both FcγRIIa and FcγRIIb as expected (Figure 1). Human mast cells from both humanized mice and human skin expressed low levels of the high-affinity IgG receptor FcγRI (CD64), but not the low-affinity IgG receptor FcγRIII (CD16) (Figure S2 in Supplementary Material). Human basophils expressed FcγRIII as previously reported (data not shown) (41).

**Figure 2:**
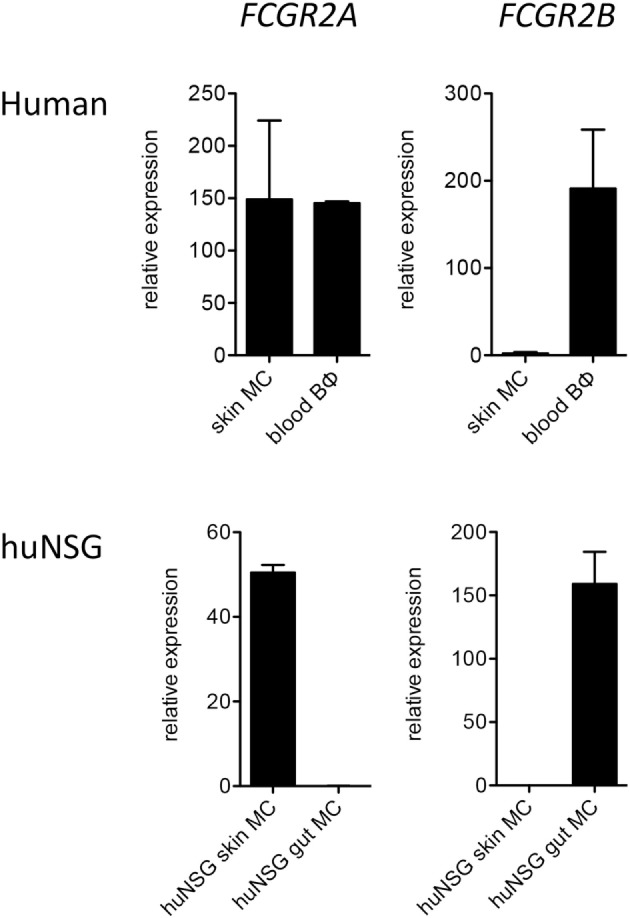
Quantitative PCR analysis of *FCGR2A* and *FCGR2B* transcripts in human FceRI^+^ cells. Data are from 5 to 7 samples per cell type.

In order to corroborate our observations we additionally assessed FcγR mRNA expression by quantitative PCR. Consistent with the flow cytometry results, mRNA for FCGR2B was present at reasonably high levels in human mast cells from humanized mouse intestine but was undetectable or nearly so in both human and humanized mouse skin mast cells (Figure 2).

**Figure 3:**
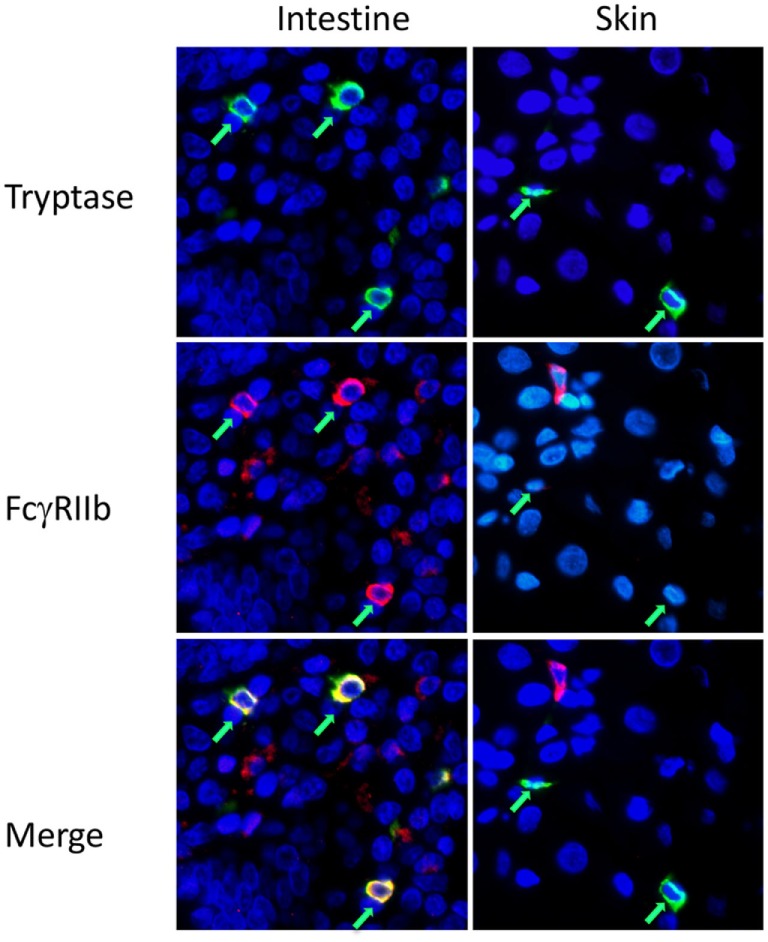
Fcγ receptor (FcγR)IIb expression by mast cells in human intestine and skin. Immunofluorescent staining for mast cell tryptase (green) and FcgRIIb (red) on human intestinal or skin tissue sections in tissue arrays. Mast cells are indicated by green arrows. Larger fields, showing more cells, are presented in Figure S3 in Supplementary Material.

For analysis of mast cell FcγR expression in a fully human setting we performed immunofluorescence analysis on normal human tissue arrays, single slides containing FFPE sections from multiple organs, processed in one pass to ensure consistency of staining and reading. FcγRIIb costaining was examined on tryptase-positive cells in human skin and jejunum. FcγRIIb was evident on the mast cells in the intestine, but not the skin (Figure 3; Figure S3 in Supplementary Material). Further analysis of the human oral-gastrointestinal tract revealed expression of FcγRIIb in mast cells of the tongue, esophagus, and both small and large intestines (Figure S4 in Supplementary Material and data not shown). Expression was lower in stomach mast cells. Staining was minimal or absent in the abundant mast cells residing in tonsils and the rare mast cells of the lymph nodes and spleen (Figure S4 in Supplementary Material and data not shown).

These analyses of mast cells in normal tissues further support the hypothesis that FcγRIIb expression by mast cells depends on the tissue context, with good expression in the gastrointestinal tract and lack of the receptor in mast cells residing in the skin, spleen, or lymph nodes.

### FcγRIIb Inhibits Human Mast Cell Activation

**Figure 4:**
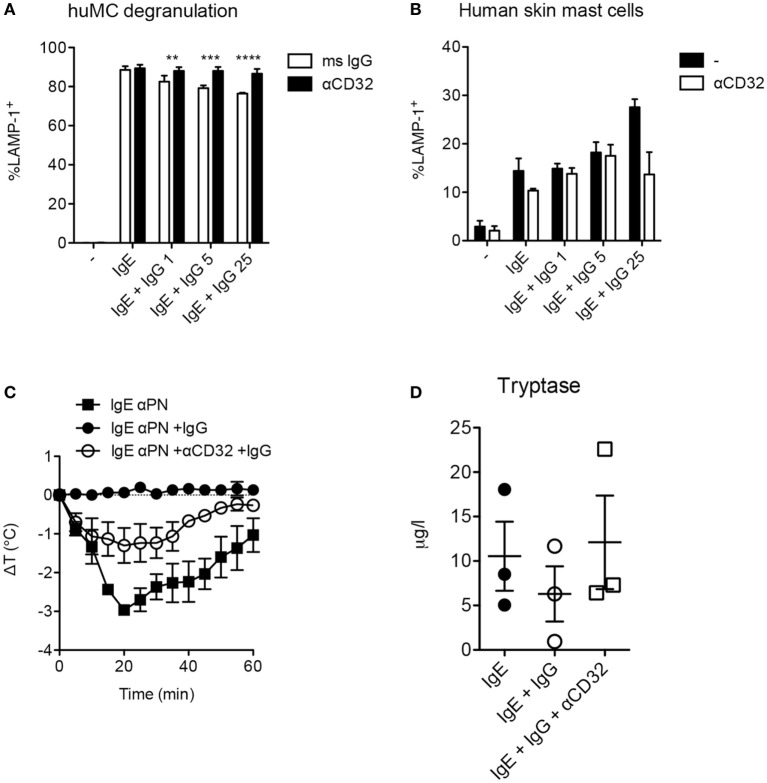
Fcγ receptor II-dependent inhibition of human mast cells and anaphylaxis. **(A)** *In vitro* degranulation of anti-PN IgE-sensitized mast cells from humanized mice and inhibition by IgG anti-PN. Anti-CD32 or an isotype control mouse IgG was added 2 h prior to IgG anti-PN. **(B)** *In vitro* degranulation of human skin mast cells under the conditions used in **(A)**. **(C)** Core temperature loss (anaphylaxis) in humanized mice passively sensitized with IgE anti-PN and challenged by PN injection. **(D)** Serum tryptase levels 1 h post-challenge in humanized mice.

In murine mast cells and human basophils IgG signals delivered *via* FcγRIIb potently suppress IgE:FcεRI-mediated activation. IgG:FcγRIIb-mediated inhibition has similarly been demonstrated in human cord blood cells, but these have an unusual phenotype, expressing only FcγRIIb but not FcγRIIa (25). We therefore sought to test for FcγRII affected IgG-mediated suppression in human mast cells expressing a complement of FcγRs more representative of normal gastrointestinal tissue resident mast cells. Using human mast cells cultivated from the bone marrow of humanized mice, we measured degranulation induced by peanut following sensitization with IgG-depleted sera from peanut allergic patients as a source of peanut-specific IgE. Sensitized mast cells rapidly upregulated LAMP-1, a marker of granule fusion following peanut stimulation (Figure 4A). The addition of IgG containing high titer anti-peanut antibodies partially reduced degranulation in a dose-responsive manner. This inhibition was fully reversed by the addition of anti-CD32 antibodies, consistent with an FcγRIIb-mediated effect. In contrast, primary human skin mast cells, which lack FcγRIIb, were not inhibited by the same concentrations of IgG (Figure 4B). In the presence of high levels of IgG, skin mast cells actually showed trends toward increased activation, with reversion by anti-CD32, suggesting that activating signals delivered *via* FcγRIIa might enhance degranulation in the absence of FcγRIIb.

As humanized mast cell FcγRIIb expression patterns reflect those in native human tissues with presence of the receptor in gastrointestinal mast cells and absence in the skin, we reasoned that this would be a useful model system in which to assess functional consequences of FcγRIIb ligation *in vivo*. We therefore used these mice to test the impact of IgG and FcγRIIb on IgE-mediated anaphylactic shock driven by human immune cells. Passive immunization of humanized mice with IgE from peanut-allergic sera sensitized them for anaphylactic shock when subsequently injected with peanut (Figure 4C). Shock severity was monitored by loss of core body temperature (loss of approximately 2.5°C) and corroborated by serum tryptase levels (Figures 4C,D). Administration of IgG containing high levels of anti-PN antibodies reduced the maximal temperature loss to around 1°C, with a non-significant trend for reduced tryptase levels. Neutralization of CD32 (FcγRIIa and FcγRIIb) partially abrogated the inhibitory effect of IgG on anaphylaxis, consistent with functional blockade of FcγRIIb-mediated suppression (Figure 4C). This suggests that the *in vivo* inhibitory effect of IgG might be mediated in part by steric blockade of antigen:IgE interaction which would not be susceptible to modulation by CD32. It is also possible that CD32 antibodies are not as effective *in vivo* because of competition for their binding by the many other CD32^+^ cells in circulation.

## Discussion

Mast cells play diverse roles in the pathophysiology of allergy, serving not only to produce the mediators of immediate hypersensitivity reactions but also as sources of cytokines that shape emerging adaptive immune responses in mucosal tissues and skin (34, 42, 43). The role of IgE antibodies in triggering their activation is well established and a number of recent observations suggest that signals delivered by IgG antibodies serve to restrain this activation by IgE. The inhibitory FcγR, FcγRIIb, plays a key role in this process. Here, we demonstrate that its expression by mast cells varies in relation to their tissue location. Our findings reveal that the inhibitory receptor is strongly expressed in tissues of the gastrointestinal tract but absent in the skin. Using a humanized mouse model, we demonstrate that allergen-specific IgG antibodies potently block IgE-mediated anaphylaxis. These findings provide important insights into the contributions of inhibitory IgG signaling in human mast cells as well as the tissue specificity of expression of the responsible receptor.

Our observations provide a potential explanation for uncertainty that has emerged in the literature regarding the relative contributions of FcγRIIb in suppressing hypersensitivity in mice versus humans. The reported absence of the receptor on primary mast cells obtained from human skin raised questions regarding its physiologic relevance in regulating their activation in humans (26). In this report, we confirm not only the absence of FcγRIIb on dermal mast cells but also clearly demonstrate its presence on human mast cells residing in the gastrointestinal tract. Several independent lines of evidence are provided to support this conclusion, including flow cytometric analysis of mast cells obtained from the tissues of humanized mice, RNA quantification of FcγRIIb mRNA in the same cells and immunofluorescence staining of FcγRIIb in tissue arrays representing a range of human organs. The fully consistent demonstration of FcγRIIb expression on gut mast cells by these three approaches lends strong support to the concept that gastrointestinal mast cells preferentially express the receptor.

The absence of FcγRIIb on dermal mast cells may additionally provide an explanation for several common clinical observations in subjects with food allergy. Patients who successfully complete OIT protocols consistently exhibit sharp increases in food allergen-specific IgG titers with only modest declines in IgE levels (44, 45). While able to tolerate enteral challenge, they often retain positive allergen skin test reactivity consistent with a scenario in which IgE-mediated activation of gastrointestinal but not dermal mast cells is suppressed by IgG:FcγRIIb signaling. We also note that in oral food challenge of allergic subjects cutaneous reactions including hives are considerably more common than gastrointestinal reactions (2).

As the role of allergen-specific IgG antibodies in respiratory and food allergy comes into sharper focus, greater understanding of the distribution and function of the receptors mediating their effects is needed. Our report provides a contribution to the field, describing the tissue-specific and cytokine-regulated heterogeneity of FcγR expression and the relationship of receptor expression patterns with observations regarding IgG-mediated suppression of allergic responses. Future studies will be needed to determine the tissue-specific factors that determine local mast cell phenotypes as well as the mast cell intrinsic mechanisms for sensing these factors. All these pathways might ultimately act as checkpoints for the allergic response and as potential targets for therapeutic intervention.

## Ethics Statement

All animal work was conducted under protocols approved by the Institutional Animal Care and Use Committee at Boston Children’s Hospital.

## Author Contributions

OB, AE, MF, SM, AS, and HO designed experiments, interpreted the results, and wrote the paper. JT and RC provided fresh human skin samples and participated in the data interpretation and reporting of the results from these samples. MR provided human tissue arrays and participated in the design and interpretation of the immunofluorescence studies.

## Conflict of Interest Statement

The authors declare that the research was conducted in the absence of any commercial or financial relationships that could be construed as a potential conflict of interest. The handling Editor declared a shared affiliation, though no other collaboration, with several of the authors OB, AE, MF, SM, AS, JT, RA, HO, and the reviewer KN declared a shared affiliation, though no other collaboration, with one of the authors MR to the handling Editor.

## References

[1] Sampson HA Albergo R. Comparison of results of skin tests, RAST, and double-blind, placebo-controlled food challenges in children with atopic dermatitis. J Allergy Clin Immunol (1984) 74(1):26–33. 10.1016/0091-6749(84)90083-6 6547461

[2] Perry TT Matsui EC Conover-Walker MK Wood RA Santos AF James LK Risk of oral food challenges. J Allergy Clin Immunol (2004) 114(5):1164–8. 10.1016/j.jaci.2004.07.063 15536426

[3] Begin P Nadeau KC Diagnosis of food allergy. Pediatr Ann (2013) 42(6):102–9. 10.3928/00904481-20130522-10 23718238 PMC4161456

[4] Holt PG Strickland D Bosco A Belgrave D Hales B Simpson A Distinguishing benign from pathologic TH2 immunity in atopic children. J Allergy Clin Immunol (2016) 137(2):379–87. 10.1016/j.jaci.2015.08.044 26518094

[5] Savilahti EM Rantanen V Lin JS Karinen S Saarinen KM Goldis M Early recovery from cow’s milk allergy is associated with decreasing IgE and increasing IgG4 binding to cow’s milk epitopes. J Allergy Clin Immunol (2010) 125(6):1315–21.e9. 10.1016/j.jaci.2010.03.025 20462631 PMC3289532

[6] James LK Shamji MH Walker SM Wilson DR Wachholz PA Francis JN Long-term tolerance after allergen immunotherapy is accompanied by selective persistence of blocking antibodies. J Allergy Clin Immunol (2011) 127(2):509–516.e1–5. 10.1016/j.jaci.2010.12.1080 21281875

[7] Caubet JC Bencharitiwong R Moshier E Godbold JH Sampson HA Nowak-Wegrzyn A Significance of ovomucoid- and ovalbumin-specific IgE/IgG(4) ratios in egg allergy. J Allergy Clin Immunol (2012) 129(3):739–47. 10.1016/j.jaci.2011.11.053 22277199

[8] Jones SM Pons L Roberts JL Scurlock AM Perry TT Kulis M Clinical efficacy and immune regulation with peanut oral immunotherapy. J Allergy Clin Immunol (2009) 124(2):292–300, 300.e1–97. 10.1016/j.jaci.2009.05.022 19577283 PMC2725434

[9] Vickery BP Pons L Kulis M Steele P Jones SM Burks AW. Individualized IgE-based dosing of egg oral immunotherapy and the development of tolerance. Ann Allergy Asthma Immunol (2010) 105(6):444–50. 10.1016/j.anai.2010.09.030 21130382 PMC3026291

[10] Kim EH Bird JA Kulis M Laubach S Pons L Shreffler W Sublingual immunotherapy for peanut allergy: clinical and immunologic evidence of desensitization. J Allergy Clin Immunol (2011) 127(3):640–6.e1. 10.1016/j.jaci.2010.12.296 21281959 PMC3052379

[11] Bedoret D Singh AK Shaw V Hoyte EG Hamilton R DeKruyff RH Changes in antigen-specific T-cell number and function during oral desensitization in cow’s milk allergy enabled with omalizumab. Mucosal Immunol (2012) 5(3):267–76. 10.1038/mi.2012.5 22318492 PMC3328586

[12] Ford LS Bloom KA Nowak-Wegrzyn AH Shreffler WG Masilamani M Sampson HA. Basophil reactivity, wheal size, and immunoglobulin levels distinguish degrees of cow’s milk tolerance. J Allergy Clin Immunol (2013) 131(1):180–6.e1–3. 10.1016/j.jaci.2012.06.003 22819512 PMC3493710

[13] Santos AF Douiri A Becares N Wu SY Stephens A Radulovic S Basophil activation test discriminates between allergy and tolerance in peanut-sensitized children. J Allergy Clin Immunol (2014) 134(3):645–52. 10.1016/j.jaci.2014.04.039 25065721 PMC4164910

[14] Du Toit G Roberts G Sayre PH Bahnson HT Radulovic S Santos AF Randomized trial of peanut consumption in infants at risk for peanut allergy. N Engl J Med (2015) 372(9):803–13. 10.1056/NEJMoa1414850 25705822 PMC4416404

[15] Daeron M Malbec O Latour S Arock M Fridman WH. Regulation of high-affinity IgE receptor-mediated mast cell activation by murine low-affinity IgG receptors. J Clin Invest (1995) 95(2):577–85. 10.1172/JCI117701 7860741 PMC295517

[16] Takai T Ono M Hikida M Ohmori H Ravetch JV van de Rijn M Augmented humoral and anaphylactic responses in Fc gamma RII-deficient mice. Nature (1996) 379(6563):346–9. 10.1038/379346a0 8552190

[17] Strait RT Morris SC Finkelman FD. IgG-blocking antibodies inhibit IgE-mediated anaphylaxis in vivo through both antigen interception and Fc gamma RIIb cross-linking. J Clin Invest (2006) 116(3):833–41. 10.1172/JCI25575 16498503 PMC1378186

[18] Burton OT Logsdon SL Zhou JS Medina-Tamayo J Abdel-Gadir A Noval Rivas M Oral immunotherapy induces IgG antibodies that act through FcgammaRIIb to suppress IgE-mediated hypersensitivity. J Allergy Clin Immunol (2014) 134(6):1310–7.e6. 10.1016/j.immuni.2014.05.017 25042981 PMC4261076

[19] Epp A Hobusch J Bartsch YC Petry J Lilienthal GM Koeleman CAM Sialylation of IgG antibodies inhibits IgG-mediated allergic reactions. J Allergy Clin Immunol (2018) 141(1):399–402.e8. 10.1016/j.jaci.2017.06.021 28728998 PMC5758435

[20] Zha L Leoratti FMS He L Mohsen MO Cragg M Storni F An unexpected protective role of low-affinity allergen-specific IgG through the inhibitory receptor FcgammaRIIb. J Allergy Clin Immunol (2018) 177(1):694–701. 10.1016/j.jaci.2017.09.054 29391255

[21] Malbec O Daeron M. The mast cell IgG receptors and their roles in tissue inflammation. Immunol Rev (2007) 217:206–21. 10.1111/j.1600-065X.2007.00510.x 17498061

[22] Nimmerjahn F Ravetch JV. Fcgamma receptors as regulators of immune responses. Nat Rev Immunol (2008) 8(1):34–47. 10.1038/nri2206 18064051

[23] Bruhns P Jonsson F. Mouse and human FcR effector functions. Immunol Rev (2015) 268(1):25–51. 10.1111/imr.12350 26497511

[24] Beutier H Gillis CM Iannascoli B Godon O England P Sibilano R IgG subclasses determine pathways of anaphylaxis in mice. J Allergy Clin Immunol (2017) 139(1):269–80.e7. 10.1016/j.jaci.2016.03.028 27246523 PMC5081282

[25] Kepley CL Taghavi S Mackay G Zhu D Morel PA Zhang K Co-aggregation of FcgammaRII with FcepsilonRI on human mast cells inhibits antigen-induced secretion and involves SHIP-Grb2-Dok complexes. J Biol Chem (2004) 279(34):35139–49. 10.1074/jbc.M404318200 15151996

[26] Zhao W Kepley CL Morel PA Okumoto LM Fukuoka Y Schwartz LB. Fc gamma RIIa, not Fc gamma RIIb, is constitutively and functionally expressed on skin-derived human mast cells. J Immunol (2006) 177(1):694–701. 10.4049/jimmunol.177.1.694 16785568 PMC2176083

[27] Burks AW Jones SM Wood RA Fleischer DM Sicherer SH Lindblad RW Oral immunotherapy for treatment of egg allergy in children. N Engl J Med (2012) 367(3):233–43. 10.1056/NEJMoa1200435 22808958 PMC3424505

[28] Begin P Winterroth LC Dominguez T Wilson SP Bacal L Mehrotra A Safety and feasibility of oral immunotherapy to multiple allergens for food allergy. Allergy Asthma Clin Immunol (2014) 10(1):1. 10.1186/1710-1492-10-1 24428859 PMC3913318

[29] Syed A Garcia MA Lyu SC Bucayu R Kohli A Ishida S Peanut oral immunotherapy results in increased antigen-induced regulatory T-cell function and hypomethylation of forkhead box protein 3 (FOXP3). J Allergy Clin Immunol (2014) 133(2):500–10. 10.1016/j.jaci.2013.12.1037 24636474 PMC4121175

[30] Vickery BP Scurlock AM Kulis M Steele PH Kamilaris J Berglund JP Sustained unresponsiveness to peanut in subjects who have completed peanut oral immunotherapy. J Allergy Clin Immunol (2014) 133(2):468–75. 10.1016/j.jaci.2013.11.007 24361082 PMC3960331

[31] Santos AF James LK Bahnson HT Shamji MH Couto-Francisco NC Islam S IgG4 inhibits peanut-induced basophil and mast cell activation in peanut-tolerant children sensitized to peanut major allergens. J Allergy Clin Immunol (2015) 135(5):1249–56. 10.1016/j.jaci.2015.01.012 25670011 PMC4418748

[32] Takagi S Saito Y Hijikata A Tanaka S Watanabe T Hasegawa T Membrane-bound human SCF/KL promotes in vivo human hematopoietic engraftment and myeloid differentiation. Blood (2012) 119(12):2768–77. 10.1182/blood-2011-05-353201 22279057 PMC3327455

[33] Burton OT Stranks AJ Tamayo JM Koleoglou KJ Schwartz LB Oettgen HC. A humanized mouse model of anaphylactic peanut allergy. J Allergy Clin Immunol (2017) 139(1):314–22.e9. 10.1016/j.jaci.2016.04.034 27417025 PMC5145786

[34] Burton OT Darling AR Zhou JS Noval-Rivas M Jones TG Gurish MF Direct effects of IL-4 on mast cells drive their intestinal expansion and increase susceptibility to anaphylaxis in a murine model of food allergy. Mucosal Immunol (2013) 6(4):740–50. 10.1038/mi.2012.112 23149659 PMC3600405

[35] Hsu FD Nielsen TO Alkushi A Dupuis B Huntsman D Liu CL Tissue microarrays are an effective quality assurance tool for diagnostic immunohistochemistry. Mod Pathol (2002) 15(12):1374–80. 10.1097/01.MP.0000039571.02827.CE 12481020

[36] van de Rijn M Gilks CB Hsu FD Nielsen TO Alkushi A Dupuis B Applications of microarrays to histopathology. Histopathology (2004) 44(2):97–108. 10.1111/j.1365-2559.2004.01766.x 14764053

[37] Daeron M Negative regulation of mast cell activation by receptors for IgG. Int Arch Allergy Immunol (1997) 113(1–3):138–41. 10.1159/000237528 9130504

[38] Cassard L Jonsson F Arnaud S Daeron M Caubet JC Bencharitiwong R Fcgamma receptors inhibit mouse and human basophil activation. J Immunol (2012) 189(6):2995–3006. 10.4049/jimmunol.1200968 22908332

[39] Ito R Takahashi T Katano I Kawai K Kamisako T Ogura T Establishment of a human allergy model using human IL-3/GM-CSF-transgenic NOG mice. J Immunol (2013) 191(6):2890–9. 10.4049/jimmunol.1203543 23956433

[40] Bryce PJ Falahati R Kenney LL Leung J Bebbington C Tomasevic N Humanized mouse model of mast cell-mediated passive cutaneous anaphylaxis and passive systemic anaphylaxis. J Allergy Clin Immunol (2016) 138(3):769–79. 10.1016/j.jaci.2016.01.049 27139822 PMC5014665

[41] Daeron M Innate myeloid cells under the control of adaptive immunity: the example of mast cells and basophils. Curr Opin Immunol (2016) 38:101–8. 10.1016/j.coi.2015.12.004 26745401

[42] Bryce PJ Miller ML Miyajima I Tsai M Galli SJ Oettgen HC Immune sensitization in the skin is enhanced by antigen-independent effects of IgE oral immunotherapy for treatment of egg allergy in children. Immunity (2004) 20(4):381–92. 10.1016/S1074-7613(04)00080-9 15084268

[43] Galli SJ Tsai M. IgE and mast cells in allergic disease. Nat Med (2012) 18(5):693–704. 10.1038/nm.2755 22561833 PMC3597223

[44] Aalberse RC van der Gaag R van Leeuwen J. Serologic aspects of IgG4 antibodies. I. Prolonged immunization results in an IgG4-restricted response. J Immunol (1983) 130(2):722–6. 6600252

[45] Akdis M Akdis CA. Mechanisms of allergen-specific immunotherapy: multiple suppressor factors at work in immune tolerance to allergens. J Allergy Clin Immunol (2014) 133(3):621–31. 10.1016/j.jaci.2013.12.1088 24581429

