# Supplementary material for: Tissue-Specific Expression of the Low-Affinity IgG Receptor, FcγRIIb, on Human Mast Cells
Source: Front Immunol. 2018 Jun 6;9:1244. doi: 10.3389/fimmu.2018.01244 (PMC5997819; doi:10.3389/fimmu.2018.01244)
Supplement: Supplementary file 1 [file Image_1.PDF]

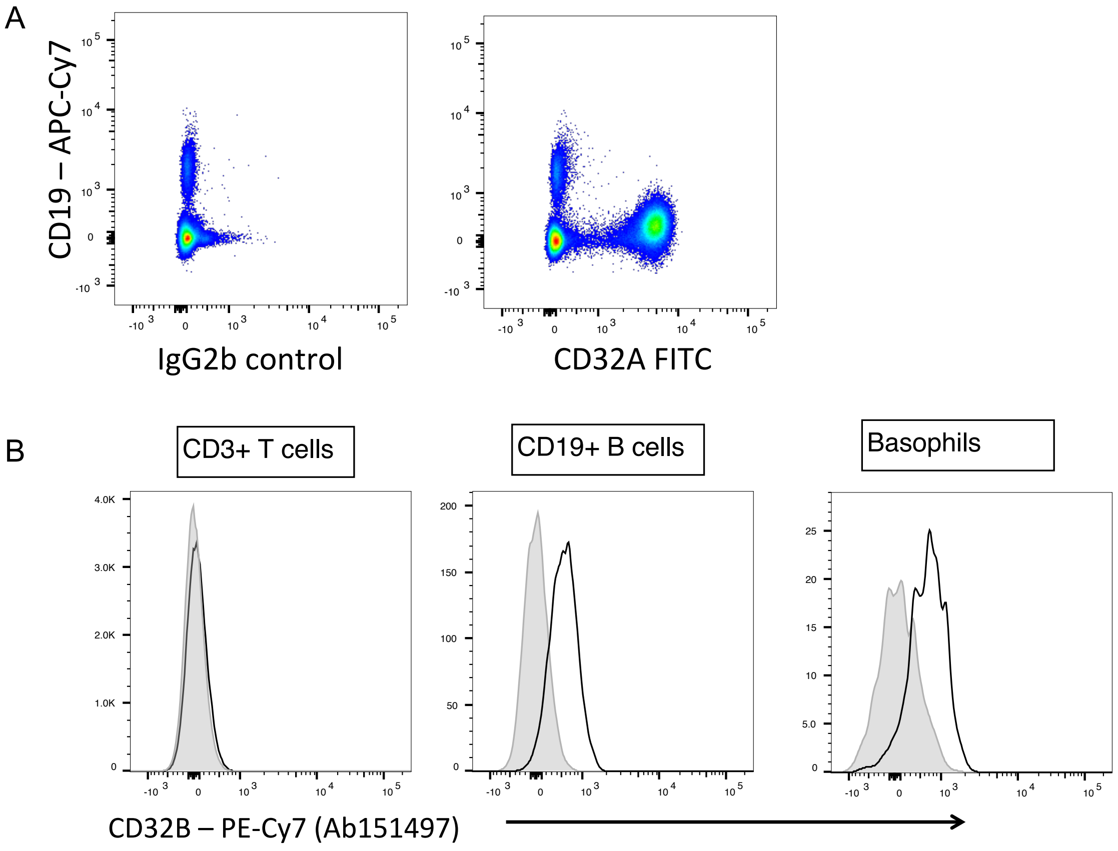

Figure S1. *Flow cytometric staining to detect FcγRIIa or FcγRIIb.* A) Staining of human peripheral blood cells for FcγRIIA using clone IV.3. Staining is absent on CD19^{+} B cells, or when a non-specific IgG2b control antibody is used. B) Intracellular staining of human peripheral blood cells for FcγRIIb using peptide-specific Ab151497 conjugated to PE-Cy7. Staining is present on B cells and basophils but not T cells. Shaded gray area indicates background fluorescence obtained when staining with PE-Cy7-conjugated non-specific rabbit IgG.

Figure S1
