# Supplementary material for: Tissue-Specific Expression of the Low-Affinity IgG Receptor, FcγRIIb, on Human Mast Cells
Source: Front Immunol. 2018 Jun 6;9:1244. doi: 10.3389/fimmu.2018.01244 (PMC5997819; doi:10.3389/fimmu.2018.01244)
Supplement: Supplementary file 2 [file Image_2.PDF]

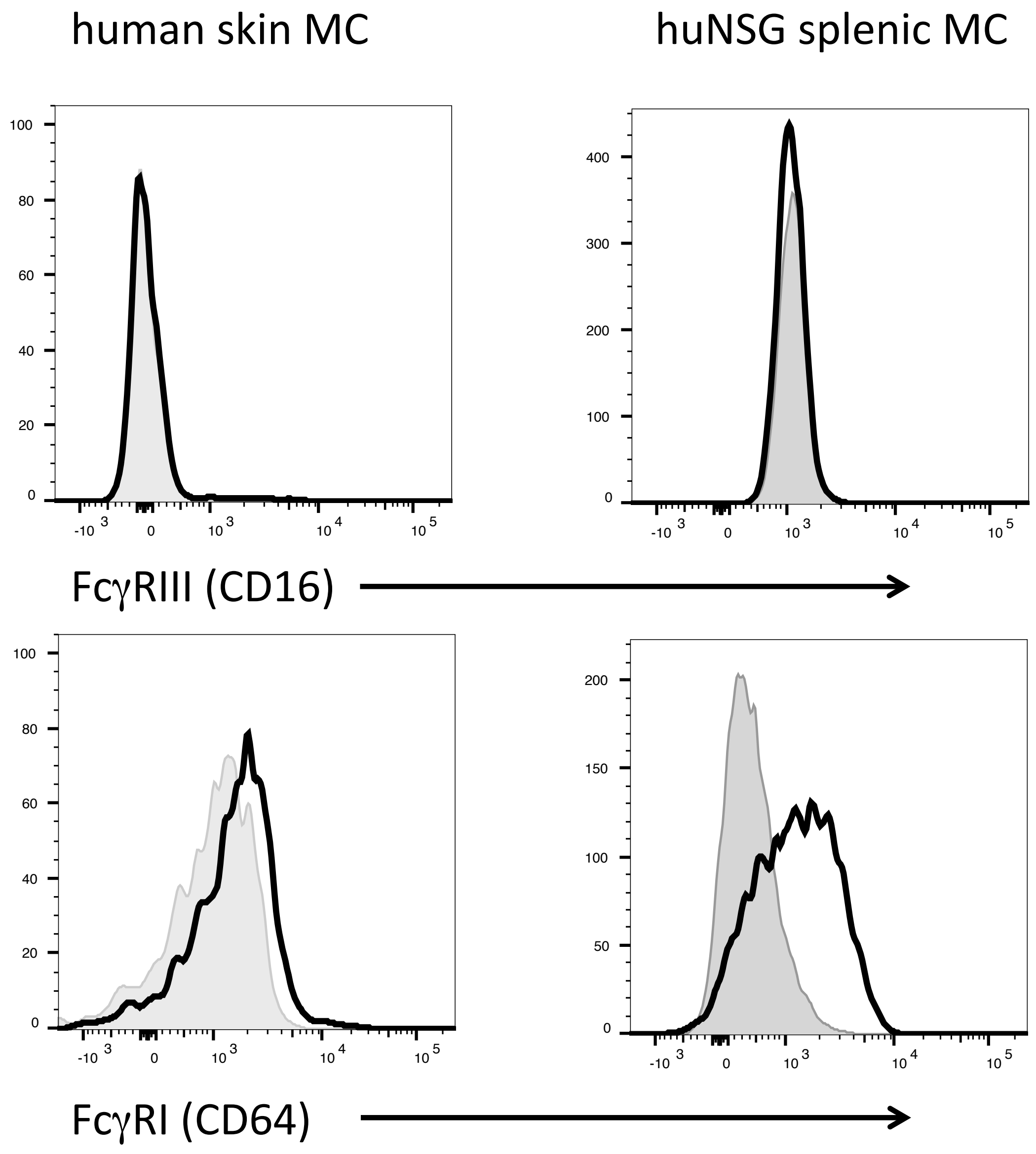

Figure S2. *Expression of FcγRI and FcγRIII on human mast cells.* Shaded gray indicates isotype control background while the solid black line indicates specific staining. Plots are representative of two experiments.

Figure S2
