# Supplementary material for: Tissue-Specific Expression of the Low-Affinity IgG Receptor, FcγRIIb, on Human Mast Cells
Source: Front Immunol. 2018 Jun 6;9:1244. doi: 10.3389/fimmu.2018.01244 (PMC5997819; doi:10.3389/fimmu.2018.01244)
Supplement: Supplementary file 3 [file Image_3.PDF]

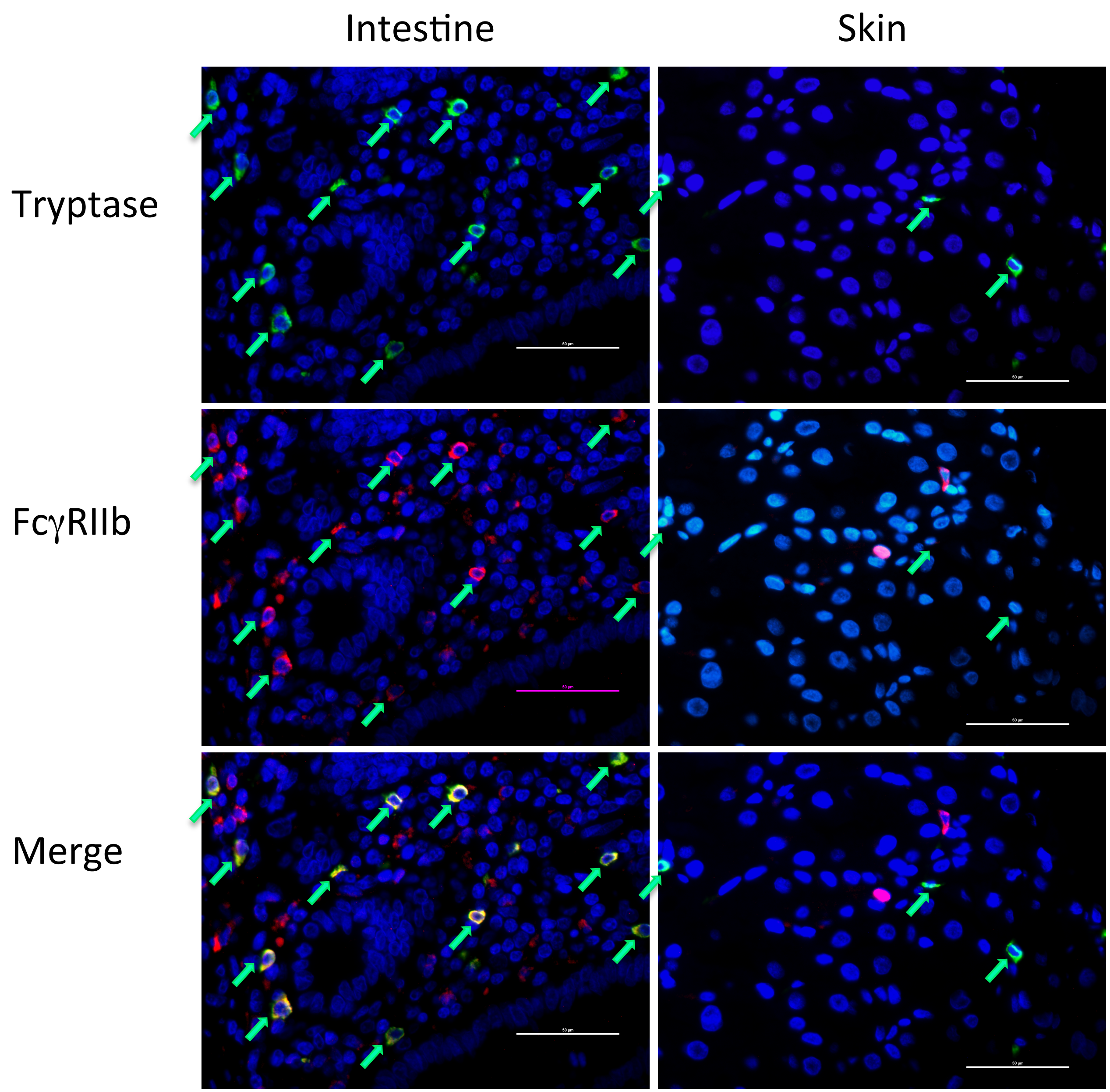

Figure S3

Figure S3. *FcγRIIb expression by mast cells in human intestine and skin.* Expanded fields from the same sections shown in **Fig 3**. Immunofluorescent staining for mast cell tryptase (green) and FcγRIIb (red) on human intestinal or skin tissue sections in tissue arrays. Mast cells are indicated by green arrows.
