# Supplementary material for: Tissue-Specific Expression of the Low-Affinity IgG Receptor, FcγRIIb, on Human Mast Cells
Source: Front Immunol. 2018 Jun 6;9:1244. doi: 10.3389/fimmu.2018.01244 (PMC5997819; doi:10.3389/fimmu.2018.01244)
Supplement: Supplementary file 4 [file Image_4.PDF]

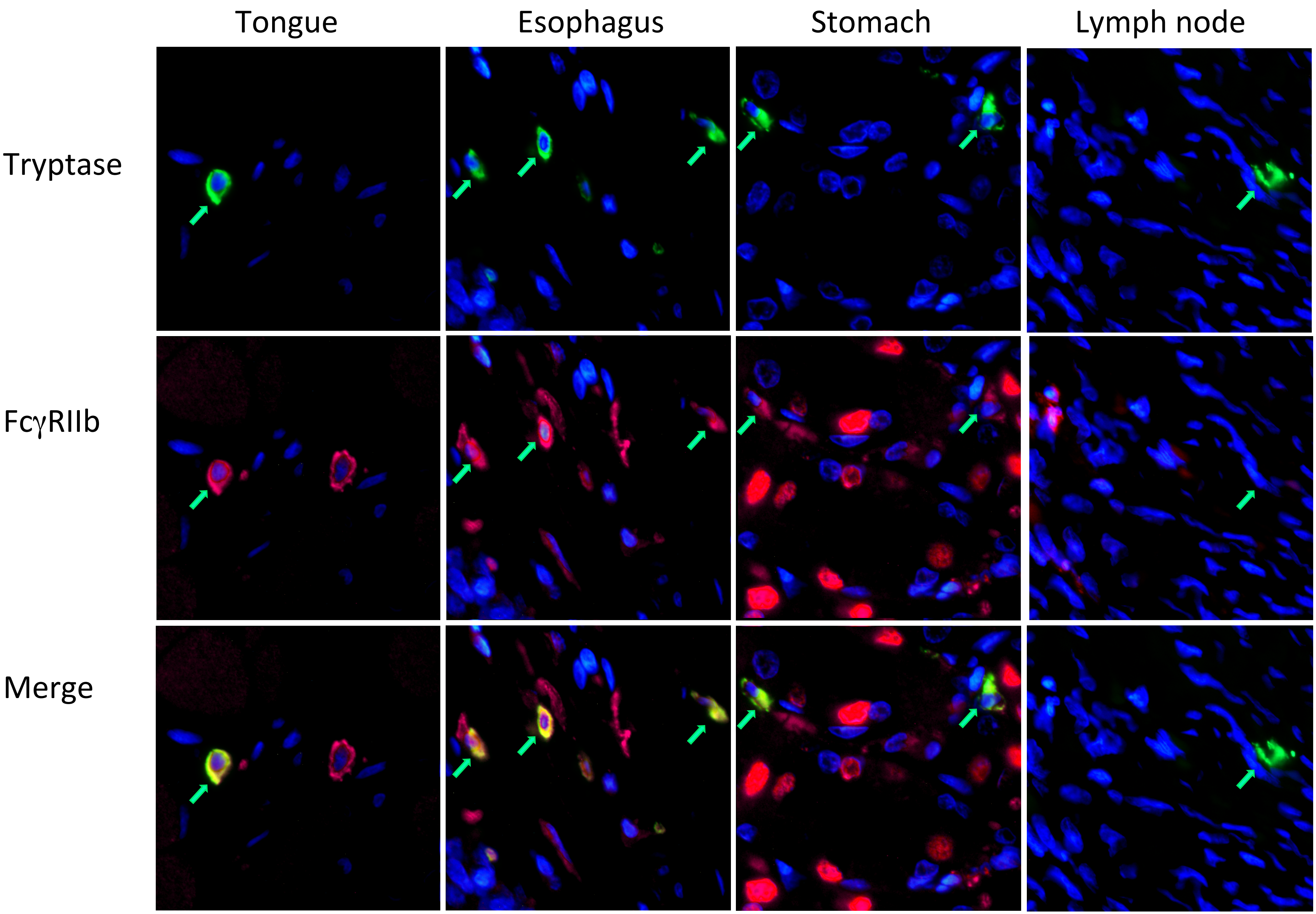

Figure S4

Figure S4. *FcγRIIb expression by mast cells in human tongue, esophagus, stomach and lymph node.* Immunofluorescent staining for mast cell tryptase (green) and FcγRIIb (red) in human tissue arrays. Mast cells are indicated by green arrows.
